# The implication of gut microbiota in recovery from gastrointestinal surgery

**DOI:** 10.3389/fcimb.2023.1110787

**Published:** 2023-02-28

**Authors:** Zhipeng Zheng, Yingnan Hu, Jingyi Tang, Wenjun Xu, Weihan Zhu, Wei Zhang

**Affiliations:** Department of General Surgery, The Second Affiliated Hospital of Zhejiang Chinese Medical University, Hangzhou, China

**Keywords:** gut microbiota, colorectal cancer, recovery, gastrointestinal surgery, postoperative complications

## Abstract

Recovery from gastrointestinal (GI) surgery is often interrupted by the unpredictable occurrence of postoperative complications, including infections, anastomotic leak, GI dysmotility, malabsorption, cancer development, and cancer recurrence, in which the implication of gut microbiota is beginning to emerge. Gut microbiota can be imbalanced before surgery due to the underlying disease and its treatment. The immediate preparations for GI surgery, including fasting, mechanical bowel cleaning, and antibiotic intervention, disrupt gut microbiota. Surgical removal of GI segments also perturbs gut microbiota due to GI tract reconstruction and epithelial barrier destruction. In return, the altered gut microbiota contributes to the occurrence of postoperative complications. Therefore, understanding how to balance the gut microbiota during the perioperative period is important for surgeons. We aim to overview the current knowledge to investigate the role of gut microbiota in recovery from GI surgery, focusing on the crosstalk between gut microbiota and host in the pathogenesis of postoperative complications. A comprehensive understanding of the postoperative response of the GI tract to the altered gut microbiota provides valuable cues for surgeons to preserve the beneficial functions and suppress the adverse effects of gut microbiota, which will help to enhance recovery from GI surgery.

## 1 Introduction

Gastrointestinal (GI) surgery, including gastrectomy, sleeve gastrectomy, Roux-en-Y gastric bypass (RYGB) surgery, and colorectal resections, has a well-established role in the treatment of different diseases, such as inflammatory bowel disease, metabolic syndrome, obesity, and multiple cancers (Tarazi et al., 2022). With the advances in technique, technology, and clinical care, the prognosis of GI surgery has been improved greatly. However, recovery from GI surgery is often disturbed by unpredictable postoperative complications. After surgical reconstruction, both the GI tract and gut microbiota gradually reach a new steady state. If this new state is not achieved or lacks microbial components essential for GI health, complications such as infections and anastomotic leak (AL) can develop, in which gut microbiota is not just a local bystander but one of the main determinants.

The commensal microbiota coevolves with the host, and the host immune system learns and develops from exposure to the commensal microbiota (Littman and Pamer, 2011). Gut microbiota helps absorption by breaking down ingested carbohydrates, fatty acids, and proteins into nutrients (Kolodziejczyk et al., 2019). Commensal bacteria contribute to colonization resistance and maintain epithelial mucosal barrier function, providing a functional layer against pathogen infection, and perturbation of the gut microbiota increases the risk of infectious complications (Ducarmon et al., 2019). Infectious complication as a tipping point allows pathogenic bacteria to cause major postoperative complications, including AL, disseminated infection, or superinfection.

The perioperative events of GI surgery from preoperative preparation to the recovery phase, exposing gut microbiota to substantial environmental changes and surgical stress, cumulatively influence its composition and function. Preoperative bowel preparations are known to disrupt gut microbiota (Nagata et al., 2019; Nalluri-Butz et al., 2022). GI segment resection exposes the lumen of the bowel to oxygen and vessel ligation causes a transient local blood supply interruption (Hartman et al., 2009). The bowel lumen exposure changes the oxygen partial pressure within the anaerobic intestinal environment, leading to the community shift in obligate anaerobes and facultative anaerobes (Hartman et al., 2009). Local tissue ischemia and reperfusion induce a decrease in the relative abundance of *Lactobacillus* and an increase in *Escherichia coli* (*E. coli*) (Wang et al., 2012; Wang et al., 2013). In addition, anatomical alterations affect GI physiology. Adaptations in GI physiology after GI surgery include changes in gastric emptying, intestinal transit, bile acid metabolism, GI surface area, acidity, and secretions, which alters the habitat of gut microbiota (Steenackers et al., 2021). Gut microbiota can sense changes in the local microenvironment and modifies its population density through quorum sensing, which also contributes to the dynamic shift of microbial phenotype (Coquant et al., 2021). Prolonged changes in circumstances give rise to potentially lethal pathogens, such as *Pseudomonas aeruginosa* (*P. aeruginosa*) and *Enterococcus faecalis* (*E. faecalis*), or make health-care-associated pathogens predominate, such as *Clostridium difficile* (Zaborin et al., 2014).

**Figure 1:**
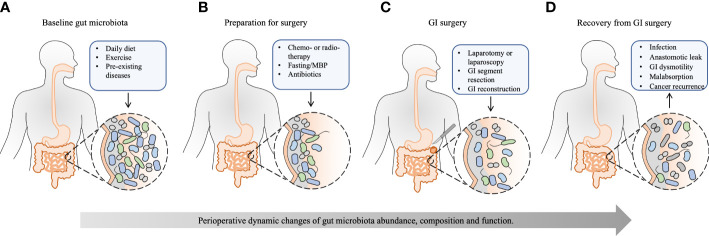
The effect of perioperative events on the gut microbiota and recovery from GI surgery. Except for the basic condition of the host, the whole process of GI surgery, from preoperative preparation to recovery from GI surgery, has a significant and accumulated effect on the gut microbiota. **(A)** The baseline gut microbiota is dependent on different daily diets, exercise, and pre-existing diseases, such as obesity, type 2 diabetes mellitus, cardio-metabolic diseases, non-alcoholic fatty liver disease, inflammatory bowel disease, and GI cancer. **(B)** Neoadjuvant chemo- or radiotherapy reduces tumor size and kills metastatic cells to facilitate the subsequent tumor removal, which may disrupt gut microbiota. Preoperative bowel preparation, including fasting, MBP, and oral antibiotics, eliminates bulky GI content and reduces the luminal bacterial load, leaving behind certain mucosal bacteria. Besides, prophylactic intravenous antibiotics have a further effect on the composition of gut microbiota. **(C)** Surgical procedures, including laparotomy or laparoscopy, GI segment resection, and subsequent GI reconstruction, change GI physiology, exert further stress on the gut microbiota, and shift bacterial phenotypes. **(D)** The healthy recovery from GI surgery is challenged by postoperative complications, such as infection, anastomotic leak, GI dysmotility, malabsorption, and cancer recurrence, which are substantially influenced by perioperative alteration of gut microbiota. Appropriate rebuilding of commensal bacteria may prevent postoperative complications and enhance postoperative recovery. MBP, mechanical bowel preparation; GI, gastrointestinal.

Therefore, postoperative rebuilding of gut microbiota is important. Inadequate or inappropriate rebuilding of gut microbiota can contribute to the development of postoperative complications. A comprehensive understanding of the interaction between host and gut microbiota is needed to enhance recovery from GI surgery. Hence, this review focuses on perioperative gut microbiota alteration and its implication in recovery from GI surgery (**Figure 1**).

## 2 Baseline gut microbiota

The baseline status of gut microbiota before surgery depends on daily diet, exercise, and chronic illness. The relationship between gut microbiota, diet, and host health is complex. Diet has an indirect effect by affecting gut microbiota composition and its production of metabolites that, in turn, can maintain health, increase the risk of disease, and be used as a treatment for certain diseases (Perler et al., 2022). Preoperative dietary modulation has been a complementary consideration among surgeons to prevent postoperative complications. A previous study using an animal model demonstrates that a short course of dietary prehabilitation with a low-fat and high-fiber diet can reverse the adverse effect of a high-fat Western-type diet (WD) on anastomotic healing *via* its effects on the gut microbiota, improving overall diversity and decreasing postoperative collagenolytic *Enterococus* (Hyoju et al., 2020). Dietary prehabilitation also improves the outcome of surgically operated mice exposed to WD diet, antibiotics, and short-term starvation (Keskey et al., 2022). Moreover, butyrate, a major product of anaerobic bacteria that has known beneficial effects on the immune system, is identified as a potential candidate biomarker to assess microbiota readiness for surgery in the context of dietary prehabilitation (Keskey et al., 2022), which helps to determine whether gut microbiota are recovered enough to support a host during stress period of preoperative treatment and surgery. Exercise as an environmental factor also has effects on gut microbiota composition, which could provide benefits to health and disease prevention. Recent studies suggest that exercise can improve the development of commensal bacteria, enrich microflora diversity, and enhance the number of beneficial microbial species (Monda et al., 2017). Meanwhile, pre-existing diseases substantially alter the composition of preoperative gut microbiota and its postoperative response to GI surgery. Increasing evidence has shown that gut microbiota is changed at the site of malignant tumors and the imbalance of gut microbiota can trigger carcinogenesis (Wong and Yu, 2019). Numerous metabolic diseases (malnutrition, obesity, type 2 diabetes mellitus, cardio-metabolic diseases, and non-alcoholic fatty liver disease), autoimmune diseases (rheumatoid arthritis, spondyloarthritis, and systemic lupus erythematosus), and living environmental and behavioral factors (alcohol and smoking) are linked to the alteration of gut microbiota and affected by it, which has been reviewed in detail elsewhere (Capurso and Lahner, 2017; Jiao et al., 2020; Fan and Pedersen, 2021).

## 3 Gut microbiota and preparation for gastrointestinal surgery

Changes in gut microbiota during preoperative treatment include both long-term chemo- or radiotherapy, not for all patients on GI surgery, and short-term just prior to surgery, such as fasting, bowel preparation, and antibiotic prophylaxis.

Neoadjuvant chemoradiotherapies for cancer can lead to compositional changes in gut microbiota. Chemotherapy drives a severe gut microbiota dysbiosis with significant decreases in the relative abundances of Firmicutes and Actinobacteria and increases in the relative abundance of Proteobacteria in patients with non-Hodgkin’s lymphoma (Montassier et al., 2015). In animal studies, 5-fluorouracil (5-Fu) reduces gut microbial community richness and diversity, leading to a decreased relative abundance of Firmicutes, Proteobacteria, Tenericutes, Cyanobacteria, and Candidate division TM7, and an increased relative abundance of Verrucomicrobia and Actinobacteria (Li et al., 2017), which is ameliorated by a probiotic mixture (Tang et al., 2017). In addition, radiotherapy reduces gut microbial diversity in patients with rising radiation enteropathy, which is significantly associated with an increased relative abundance of *Clostridium IV*, *Roseburia*, and *Phascolarctobacterium* (Reis Ferreira et al., 2019). In a mouse model, the relative abundances of phylum Proteobacteria, genera *Escherichia, Shigella* and *Eubacterium xylanophilum_group*, and species *Lactobacillus murinus* are correlated with radiation dose, and after 7 days of radiation, the diversity of gut microbiota is significantly decreased, in which the relative abundance of Proteobacteria and Bacteroides is increased, while that of Tenericutes and *Roseburia* is decreased, which also can be mitigated by administration of compound probiotics (Zhao et al., 2021). Moreover, not only can chemotherapy and radiotherapy alter gut microbiota, but gut microbiota can directly or indirectly modulate cancer response to chemotherapy, radiotherapy, and immunotherapy (Iida et al., 2013; York, 2018; Tonneau et al., 2021).

In addition, bowel preparations for major surgery instantly disrupt gut microbiota and might lead to prolonged detrimental consequences (Perez-Cobas et al., 2013; Zarrinpar et al., 2014; Jalanka et al., 2015). Physiological stress induced by surgical processes, involving skin incision, tissue dissection, organ resection, and anastomosis, can increase susceptibility to infection. Therefore, the current clinical practice of bowel preparation is used to eliminate potential pathogens as much as possible before major surgery to minimize intraoperative contamination. Daily feeding/fasting rhythms lead to daily cyclical compositional fluctuations in the gut microbiota, which contribute to gut microbial diversity and affect host metabolism (Zarrinpar et al., 2014). Unlike these daily gut microbiota fluctuations, altered jejunal microbiota with decreased abundance of Betaproteobacteria and Bacteroidales is observed in dogs subjected to a prolonged period of fasting (Kasiraj et al., 2016). MBP is generally used to eliminate bulky contents and reduce the bacterial load in the GI tract, which leaves behind certain strains of mucosa-associated bacteria and induces shifts in gut microbiota. The lavage leads to an instant and substantial change in the bacterial levels and composition of gut microbiota, specifically decreased *Bifidobacterium* and *Lactobacillus* and increased *E. coli* and *Staphylococcus*, which can be restored within 14 days, and the recovery rate is dose-dependent (Wu et al., 2012; Jalanka et al., 2015). MBP has a profound effect on the gut metabolome, but it also recovers to baseline within 14 days (Nagata et al., 2019). In contrast, MBP with oral antibiotics shapes gut microbiota composition acutely and longitudinally, which requires at least 30 days to return to a level similar to baseline (Nalluri-Butz et al., 2022). Oral or intravenous antibiotics and the local application of topical antimicrobial solutions are variably used as routine practices to disinfect the intestine and skin (Guenaga et al., 2011; Nelson et al., 2014). Oral antibiotics are often used for bowel preparation and most patients receive intravenous antibiotics at the time of GI surgery, both of which facilitate the clearance of potential pathogens, exert selective pressure on bacterial composition and function, and further perturb commensal microbial communities (Antonopoulos et al., 2009; Ubeda and Pamer, 2012; Ferrer et al., 2014). The effects of these preparations for surgery on gut microbiota preservation and bacterial phenotype shifts remain unknown and need further study.

## 4 Gut microbiota and surgical procedures

The surgery itself is the major factor affecting gut microbiota composition and function. After surgical resection of diseased, obstructed, or ischemic intestinal segments, intestinal reconstructions are generally required to reestablish intestinal continuity. These surgical reconstructions of the GI tract affect the intraluminal environment, intestinal permeability, transit time, food digestion, and nutrient absorption, but the GI tract can adapt to the new anatomical structure, which is a natural compensatory process and nearly allows patients to recover to normalcy (Carswell et al., 2014; Tappenden, 2014; Ladebo et al., 2021). It is now believed that much of food digestion and nutrient absorption depends on the action of the gut microbiota, and gut microbes use ingested nutrients for their fundamental biological processes, metabolic outputs of which have significant impacts on host physiology (Gentile and Weir, 2018). Besides, each part of the intestine has a specialized ecosystem that facilitates the breakdown, processing, and absorption of nutrients, and the surgical reconstructions of the GI tract can change downstream gut microbiota, affecting the metabolism and immune functions (Sinclair et al., 2018; Li et al., 2021). The effects of these changes on the clinical outcomes of GI surgery need further investigation.

Sleeve gastrectomy (SG) and Roux−en−Y gastric bypass (RYGB) are the most common surgical approaches to treat morbid obesity. The beneficial effects of bariatric surgery are not only contributed by stomach pouch restriction and malabsorptive configuration induced by the surgical operation itself, but changes in gut microbiota may also be part of the mechanism. Although these surgeries are not for treating specific gastrointestinal diseases in this setting, their effects on gut microbiota are now being studied. Gut microbiota is altered by surgery and the profound alterations persist in the first year of follow-up with an increase in Bacteroides and Proteobacteria and a decrease in Firmicutes in most studies (Luijten et al., 2019). The shift of gut microbiota after a bariatric surgery significantly differs between different surgical procedures. RYGB has a deeper impact on the composition and function of gut microbiota than SG (Farin et al., 2020; Salazar et al., 2022). Laparoscopic RYGB leads to a higher relative abundance of aero-tolerant bacteria (*E. coli* and *Streptococcus*), whereas anaerobes (*Clostridium*) are more abundant after SG (Farin et al., 2020). In addition, enrichment of *Akkermansia muciniphila* is observed 6 months after both surgeries (Farin et al., 2020). In another short-term study, the relative abundance of *Akkermansia*, *Eubacterium*, *Haemophilus*, and *Blautia* is higher 3 months after SG, while the relative abundance of *Veillonella*, *Slackia*, *Granucatiella*, and *Acidaminococcus* is higher after RYGB (Sanchez-Alcoholado et al., 2019). Changes in gut microbiota are an adaptive response to the altered gut environment, and specific gut microbiota signatures mediate the successful rate of bariatric surgery through their interaction with the bile acids milieu (Gutierrez-Repiso et al., 2019). Moreover, these alterations to the gut microbiota after RYGB are conserved among humans, rats, and mice, with a rapid and sustained increase in Bacteroidetes, Proteobacteria (*Escherichia*), and Verrucomicrobia (*Akkermansia*) (Liou et al., 2013). Besides, fecal microbiota transplant (FMT) from RYGB-treated mice to germ-free mice not receiving intestinal reconstruction leads to weight loss and decreased fat mass in the recipient mice compared to those that receive FMT from sham surgery mice (Liou et al., 2013), suggesting that gut microbiota after RYGB contributes to the effects of RYGB on the body weight and metabolism. These studies indicate that intestinal surgery-induced altered gut microbiota-host interactions, in turn, affect the prognosis of surgery.

Alterations of gut microbiota after gastrectomy are observed in patients with gastric cancer, showing greater species diversity and richness, higher abundance of oral microbes, aerobes (*Streptococcus* and *Enterococcus*), and facultative anaerobes (*Escherichia, Enterobacter*, and *Streptococcu*s) than control participants (Erawijantari et al., 2020). In addition, gut microbiota plays an important role in the occurrence and development of colorectal cancer (CRC) (Lin et al., 2019). It is worth noting that several CRC-related bacteria (*Fusobacterium nucleatum* and *Atopobium parvulum*) and secondary bile acids such as genotoxic deoxycholic acid are significantly enriched in patients undergoing total gastrectomy compared with the control group (Erawijantari et al., 2020). In contrast, surgery greatly reduces the diversity of the gut microbiota in CRC patients (Cong et al., 2018; Deng et al., 2018). The gut microbiota of postoperative patients and CRC patients differ significantly. The relative abundance of Proteobacteria is increased in postoperative CRC patients compared with that in preoperative CRC patients and healthy individuals, and the *Klebsiella* has a higher proportion in postoperative CRC patients than that in preoperative CRC patients, which is also significantly associated with infectious diseases and lymphatic invasion (Cong et al., 2018). Subdividing postoperative CRC patients according to the presence or absence of newly developed adenoma, the gut microbiota of patients with newly developed adenoma is different from that of clean intestine patients and is similar to the gut microbiota of carcinoma patients (Jin et al., 2019). The difference in the gut microbiota between the two groups can be used as biomarkers to distinguish postoperative patients with or without newly developed adenoma with an AUC value of 0.72 (Jin et al., 2019), suggesting that gut microbiota may be used as non-invasive biomarkers to predict newly developed adenomas and prevent cancer recurrence in postoperative patients. These changes in the gut microbiota are long-lasting and correlate with the clinical course. Especially in CRC patients with postoperative complications, the gut microbiota shows significant changes in postoperative CRC patients, which do not resolve, even 24 months after surgery (Schmitt et al., 2021). The association between long-term gut microbiota alteration and postoperative complications indicates that gut microbiota modulation may help to optimize the outcome of CRC patients after surgery.

## 5 Gut microbiota-associated complications after gastrointestinal surgery

Numerous postoperative complications, such as infections, AL, GI dysmotility, malabsorption, and cancer recurrence, continue to hinder recovery from GI surgery with complex reconstructive procedures. A better molecular understanding of the perioperative interaction between the GI tract and gut microbiota will make surgery safer and further prevent complications.

### 5.1 Infection

Postoperative infection, surgical site infections (SSIs) in particular, is the most common reason for readmission, which contributes to the increased cost of health care (Merkow et al., 2015). In general, GI surgery is subjected to a greater risk of postoperative infection compared with other surgical procedures because the GI tract is inhabited by a wide cluster of microorganisms. Traditionally, postoperative infection is often caused by inadequate preoperative topical or intestinal disinfection, which can be prevented by antibiotics as prophylaxis the day before surgery, even without mechanical bowel preparation (Espin Basany et al., 2020). However, complete depletion of gut and skin microbiota is not possible and has potential negative effects. Many postoperative infections arise from the patient’s gut microbiota, usually after unintended inhibition of beneficial bacteria and translocation of antibiotic-resistant pathogenic bacteria (Deitch, 2012; Yu et al., 2014). Conversely, a diverse and protective gut microbiota provides an important biological layer against infectious complications, because commensal bacteria contribute to colonization resistance against both endogenous and exogenous pathogens through competitive inhibition, production of antimicrobial peptides, and activation of the host immune system (Ducarmon et al., 2019; Isles et al., 2022).

Lifestyle factors, underlying diseases, medications, antibiotics, and surgical procedures can perturb gut microbiota leading to loss of colonization resistance and increased susceptibility to the invasion of pathogenic bacteria. Besides, many in−hospital infections originate from the patient’s gut microbiota when the beneficial bacterial population is suppressed (Wang et al., 2019). Preoperative dysbiosis of gut microbiota is associated with higher rates of postoperative infectious complications, including abdominal/pelvic infections and pulmonary infections, in CRC patients (Liu et al., 2021). The increased relative abundance of *Klebsiella* in postoperative CRC patients is significantly and positively associated with infectious diseases, such as bacterial invasion of epithelial cells and *Staphylococcus aureus* infection, revealed by the correlation analysis between differentiated metabolic pathways and genera (Cong et al., 2018). The causative bacteria of surgical site infections are identified to be *P. aeruginosa*, *Staphylococcus aureus*, and *Enterococcus* spp., which are also enriched in the fecal microbiota of postoperative CRC patients (Ohigashi et al., 2013). A gut microbiota-based model base on six genera (*Hungatella*, *Epulopiscium*, *Fusobacterium*, *Ruminococcaceae_ucg_009*, *Actinomyces*, and *Ralstonia*) is used to predict surgical site infections after ileocolonic resection for Crohn’s disease (CD) patients with an AUC of 0.78 (Julien et al., 2022).

The effect of perioperative modulation of gut microbiota by probiotic or synbiotic on postoperative infectious complications has been evaluated in clinical trials (**Table 1**). A meta-analysis shows that perioperative probiotic or prebiotic supplements may reduce the overall incidence of infectious complications, including wound infections, respiratory infections, and urinary tract infections, in patients undergoing GI surgery, and the most used strains are *Lactobacillus* and *Bifidobacterium* (Yang et al., 2017). Their role in postoperative infections may be due to the perioperative stabilization of gut microbiota and alleviation of the systemic inflammatory response (Kotzampassi et al., 2015; Polakowski et al., 2019). Using probiotics or prebiotics as a potential alternative to maintaining the beneficial structure of gut microbiota throughout the whole process of hospitalization may be a promising strategy to reduce the risk of postoperative infections and can be included in enhanced recovery after surgery. However, the results of existing clinical studies are inconsistent, which may be due to disparities in type, dosage, and administration strategies (timing and duration) of probiotics/prebiotics, thus large-scale randomized clinical trials are needed to confirm the efficacy and safety of probiotics/prebiotics in surgical patients.

**Table 1:** Effect of synbiotics or probiotics on infectious complications, anastomotic leak, and gastrointestinal motility after gastrointestinal surgery.

Formulation	Timing (Duration)	Effect on infectious complications	Effect on anastomotic leak	Effect on gastrointestinal motility
Synbiotics (oligofructose, *Lactobacillus* *acidophilus* La5, *Lactobacillus bulgaricus*, *Bifidobacterium lactis* BB-12 and *Streptococcus thermophilus*) (Reddy et al., 2007)	Pre (NA)	No effect	NA	NA
Synbiotics (betaglucan, inulin, pectin, resistant starch, *Pediacoccus pentosaceus* 5-33:3, *Leuconostoc mesenteroides* 32–77:1, *Lactobacillus paracasei* subsp. *paracasei* 19 and *Lactobacillus plantarum* 2362) (Horvat et al., 2010)	Pre (NA)	No effect	NA	No effect
Probiotics (*Lactobacillus plantarum*, *Lactobacillus acidophilus*, and *Bifidobacterium longum*) (Liu et al., 2011)	Pre (6 d) and Post (10 d)	Decreased central lines infection, pneumonia infection, and urinary infection	NA	Reduced first defecation time
Probiotics (*Lactobacillus plantarum* 299v) (Mangell et al., 2012)	Pre (8 d) and Post (5 d)	No effect	No effect	No effect
Probiotics (*Bifidobacterium longum*, *Lactobacillus acidophilus* and *Enterococcus faecalis*) (Zhang et al., 2012)	Pre (3 d)	Decreased bacteremia and septicemia	No effect	No effect
Probiotics (*Lactobacillus plantarum*, *Lactobacillus acidophilus*, and *Bifidobacterium longum*) (Liu et al., 2013)	Pre (6 d) and Post (10 d)	Decreased septicemia, central lines infection, pneumonia infection, and urinary infection	NA	Reduced first defecation time
Synbiotics (betaglycan, inulin, pectin, resistant starch, *Pediacoccus pentosaceus* 5-33:3, *Leuconostoc mesenteroides* 32–77:1, *Lactobacillus paracasei* subsp. *paracasei* 19 and *Lactobacillus plantarum* 2362) (Krebs et al., 2013)	Pre (3 d)	No effect	NA	No effect
Probiotics (*Bifidobacterium bifidum*) (Sadahiro et al., 2014)	Pre (7 d) and Post (10 d)	No effect	No effect	NA
Probiotics (*Lactobacillus acidophilus*, *Lactobacillus plantarum*, *Bifidobacterium lactis* and *Saccharomyces boulardii*) (Kotzampassi et al., 2015)	Pre (1 d) and Post (15 d)	Decreased surgical site infection and pneumonia infection	Reduced	Reduced first flatus and defecation time
Probiotics (*Saccharomyces boulardii*) (Consoli et al., 2016)	Pre (7 d)	No effect	NA	NA
Synbiotics (galactooligosaccharides, *Lactobacillus casei*, and *Bifidobacterium breve*) (Komatsu et al., 2016)	Pre (7–11 d) and Post (2–7 d)	No effect	No effect	NA
Probiotics (*Bifidobacterium longum* BB536) (Mizuta et al., 2016)	Pre (7–14 d) and Post (14 d)	No effect	No effect	NA
Probiotics (*Lactobacillus acidophilus*, *Lactobacillus casei*, *Lactobacillus lactis*, *Bifidobacterium bifidum*, *Bifidobacterium longum*, and *Bifidobacterium infantis*) (Tan et al., 2016)	Pre (7 d)	No effect	No effect	NA
Probiotics (*Bifidobacterium longum*, *Lactobacillus acidophilus*, and *Enterococcus faecalis*) (Yang et al., 2016)	Pre (5 d) and Post (7 d)	No effect	No effect	Reduced first flatus and defecation time
Synbiotics (fructo-oligosaccharide, *Lactobacillus acidophilus* NCFM, *Lactobacillus rhamnosus* HN001, *Lactobacillus casei* LPC-37, and *Bifidobacterium lactis* HN019) (Flesch et al., 2017)	Pre (5 d) and Post (14 d)	Decreased surgical site infection, abdominal infection, and pneumonia infection	No effect	NA
Probiotics (*Bifidobacterium* and *Lactobacillus*) (Zhao et al., 2017)	Post (7 d)	NA	NA	Reduced first flatus time
Probiotics (NA) (Xie et al., 2018)	Post (8 d)	No effect	No effect	Reduced first flatus time
Synbiotics (fructo-oligosaccharide, *Lactobacillus acidophilus* NCFM, *Lactobacillus rhamnosus* HN001, *Lactobacillus casei* LPC-37, and *Bifidobacterium lactis* HN019) (Polakowski et al., 2019)	Pre (7 d)	Decreased overall infectious complications	NA	NA
Probiotics (*Lactobacillus acidophilus*, *Lactobacillus casei*, *Lactobacillus plantarum*, *Lactobacillus rhamnosus*, *Bifidobacterium lactis*, *Bifidobacterium bifidum*, *Bifidobacterium breve*, *Streptococcu sthermophilus*) (Bajramagic et al., 2019)	Post (1 year)	No effect	No effect	Reduced postoperative ileus
Probiotics (*Bifidobacterium longum*, *Lactobacillus bulgaricus*, and *Streptococcus thermophilus*) (Xu et al., 2019)	Pre (7 d)	No effect	NA	Reduced first flatus time
Probiotics (*Bifidobacterium animalis* subsp. *lactis* HY8002, *Lactobacillus casei* HY2782, and *Lactobacillus plantarum* HY7712) (Park et al., 2020)	Pre (7 d) and Post (21 d)	No effect	NA	No effect
Probiotics (*Lactobacillus rahmnosus* GG) (Folwarski et al., 2021)	Post (30 d)	No effect	No effect	Reduced first flatus and defecation time

Pre, preoperatively; Post, postoperatively; d, days; NA, not available.

### 5.2 Anastomotic leak

Anastomotic leak (AL) is one of the most potentially devastating complications that can develop after major surgical bowl reconstruction and has plagued surgeons for decades. Although surgical technique and postoperative care have been improved over the past several decades, AL continues to occur and can lead to peritonitis, sepsis, and even death (Caulfield and Hyman, 2013; Shogan et al., 2013). After anastomosis is performed, the repairing process of the GI tract is immediately initiated and divided into three phases, including the lag phase, the fibroplasia phase, and the maturation phase (Sajid et al., 2012). Ultimately, the healing process results in the repair of the intestinal epithelial barrier with the involvement of complex molecular and cellular interactions of host cells, luminal proliferative components, and gut microbiota (Kurashima and Kiyono, 2017). Among these factors contributing to anastomotic healing, gut microbiota remains largely overlooked and should be focused on. Indeed, germ-free mice have a reduced regenerative response of the epithelia and a hampered healing process of the intestinal barrier compared with conventional mice (Hooper et al., 2001). In addition, conventionalized germ-free rats show a significantly better wound healing of intestinal anastomoses than germ-free animals and rats that are colonized by either *Lactobacillus acidophilus* or *E. coli* (Okada et al., 1999), suggesting that the effect of gut microbiota on the healing of intestinal anastomoses depends on differences in the types of bacteria. Therefore, gut microbiota can either assist or hinder intestinal wound healing through cooperation or competition between different microbial species (Koliarakis et al., 2020). Further studies focusing on gut microbiota could be one such avenue for uncovering the elusive pathogenesis of AL.

The gut microbiota participates in the physiological process of intestinal wound healing and epithelial repair through a variety of molecular mechanisms. Members of the gut microbiota can interact with different intestinal epithelial lineages through innate immune receptors, such as toll-like receptor (TLR) 4 and 2, by recognizing gut microbial components, such as lipopolysaccharide and flagellin, thus modulating homeostasis in the gut and playing an important role in the epithelial repair after injury (Burgueno and Abreu, 2020). Metabolites produced by gut microbiota are also involved in the intestinal epithelial repair. Butyrate, one of the SCFAs derived from the bacterial fermentation of dietary fibers, regulates colonocyte proliferation, strengthens the gut barrier, limits pathogen growth, and suppresses inflammatory response (Martin-Gallausiaux et al., 2021). Several animal studies have shown that exogenous butyrate administration improves the healing of colonic anastomoses and enhances colonic anastomotic strength in rats (Rolandelli et al., 1997; Mathew et al., 2010; Bosmans et al., 2017). Besides, perioperative supplementation of inulin and galactooligosaccharides, which modulate the gut microbiota to increase the production of butyrate through enhancement of butyrate-producing bacteria, improve anastomotic healing and reinforce the gut barrier in mice (Hajjar et al., 2021). Moreover, SCFAs produced by gut microbiota may be a mechanism of intestinal resistance to colonization by *P. aeruginosa*, which has been identified as an AL-related pathogen (Levison, 1973; Williamson and Alverdy, 2021). These data indicate the importance of functional gut microbiota to ensure adequate healing, but the stress of perioperative events and surgery itself may induce a shift of gut microbiota to a pathologic phenotype, which leads to an AL.

Previous studies have investigated the causal relationship between AL and gut microbiota. Anastomotic injury, without preoperative MBP or antibiotic prophylaxis, contributes to remarkable compositional and functional changes in the anastomotic tissue-associated microbiota with a 200-fold and 500-fold increase in the relative abundance of *Escherichia-Shigella* and *Enterococcus*, respectively (Shogan et al., 2014). However, these alterations were not observed in the stool microbiota (Shogan et al., 2014), suggesting that certain bacteria having adhesive properties invade the anastomotic site, which may potentially complicate or accelerate anastomotic healing.

**Figure 2:**
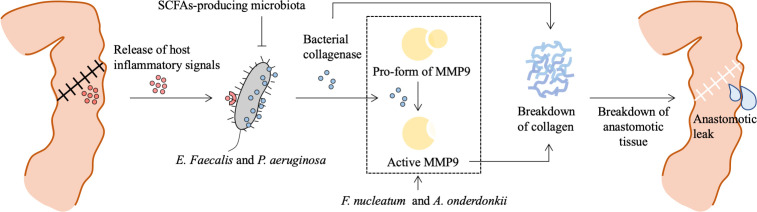
Role of bacteria in the pathogenesis of anastomotic leak. Surgical resection and reconstruction induce physiological stress and lead to a release of inflammatory signals, which contribute to phenotype transformation or selection of gut microbiota. *E. Faecalis* and *P. aeruginosa* sense and respond to the host’s local stress signals resulting in increased adherence capacity and collagenase production. Bacterial collagenase directly breaks down collagen I and indirectly breaks down collagen IV by converting local tissue MMP9 to its active form. SCFAs-producing microbiota prevents *P. aeruginosa* colonization. Besides, *F. nucleatum* can stimulate epithelial cells’ MMP9 expression contributing to the loss of submucosa collagen I and III, and *A. onderdonkii*-induced TNF-α and IL-1β production by monocytes and neutrophils are also involved in MMP9-dependent breakdown of collagen. This process results in a breakdown of anastomotic tissue and is involved in the pathogenesis of anastomotic leak. MMP9, matrix metalloproteinase 9; SCFAs, short chain fatty acids; *F. nucleatum*, *Fusobacterium nucleatum*.

Clinically relevant animal models of AL are created to investigate the responsible gut microbiota-related mechanisms involved in the pathogenesis of AL. A novel model of AL, in which rats are exposed to preoperative radiation as in patients with advanced rectal cancer and undergo a distal colon resection followed by intestinal inoculation with *P. aeruginosa*, a common pathogen in the gut following radiation exposure, is developed (Olivas et al., 2012). Only those rats that are both exposed to radiation and colonized by *P. aeruginosa* occur AL. Sequencing of retrieved *P. aeruginosa* strains from the leaking anastomotic tissues demonstrates that the appearance of a single nucleotide polymorphism mutation in the mexT gene leads to a stop codon and subsequent a non-functional truncated protein, which induces *P. aeruginosa in vivo* transformation to more destructive phenotype with enhanced abilities of collagen degradation, invasion, and cytotoxicity. This study indicates that interactions between pathogen and host stimulate and contribute to the development of bacterial virulence and subsequent AL. More molecular detail on the microbiota-dependent pathogenesis of AL also has been identified. A commensal bacterium, *E. faecalis*, contributes to the pathogenesis of AL by degrading collagen and activating host matrix metalloproteinase 9 (MMP9) (Shogan et al., 2015). Rat receiving a low colonic reconstruction and segmental devascularization develop a 50% incidence of AL. The leaking anastomotic tissues are colonized by *E. faecalis* strains with high collagenase activity. In contrast, the healed anastomotic tissues harbor low collagenase *E. faecalis* strains. Both eliminations of *E. faecalis* strains by direct topical antibiotics and pharmacological suppression of intestinal MMP9 activation prevent AL. Mechanically, high collagenase-producing strains directly cleave collagen I and indirectly break down collagen IV by converting tissue MMP9 to its active form, which is dependent on the collagenase-encoding genes *gelE* and *sprE*. *Fusobacterium nucleatum* (*F. nucleatum*) also can induce colon AL by activating epithelial cells to express MMP9 (Shi et al., 2022). Recent studies using FMT demonstrate a causal role of the preoperative and postoperative gut microbiota in AL. FMT using postoperative stool samples from AL patients leads to poor anastomotic healing in rats (Jin et al., 2022). Mice receiving preoperative stool samples from AL patients with CRC also display poor surgical healing, which is correlated with two bacterial strains, *Alistipes onderdonki* (*A. onderdonkii*) and *Parabacteroides goldsteinii* (*P. goldsteinii*), and *A. onderdonkii* supplementation leads to a higher rate of leaks in mice (Hajjar et al., 2022). Overall, some individual pathogens, including *E. faecalis*, *P. aeruginosa*, *F. nucleatum*, and *A. onderdonkii*, have been implicated in the pathogenesis of AL by promoting the breakdown of collagen (**Figure 2**). A short course of dietary prehabilitation decreases postoperative collagenolytic *Enterococcus* and reduces AL in mice (Hyoju et al., 2020). Although the effect of probiotics or synbiotics on the AL is not satisfactory in most clinical cases according to limited reports (**Table 1**), *P. goldsteinii* plays an anti-inflammatory effect and improves wound healing in a murine AL model (Hajjar et al., 2022), representing a potential probiotic that may enhance postoperative recovery in patients at risk of developing AL.

Taken together, these animal data suggest that bacterial phenotypes can be reshaped *in vivo* by injured tissue, which may have a profound effect on the recovery from GI surgery. Targeting the gut microbiota as a modifiable factor may be a novel strategy for the prevention of AL.

### 5.3 Gastrointestinal dysmotility

GI dysmotility is common after major GI reconstruction. This dysmotility can range from malabsorption, which is associated with increased motility, to postoperative ileus (POI). Gut microbiota plays a significant role in the regulation of GI physiology, particularly GI motility (Zheng et al., 2022).

Altered gut microbiota after GI surgery substantially contributes to changes in GI motility. POI is one of the most frequent complications after GI surgery. Mice treated with oral antibiotics show a moderate alleviation in small intestinal POI, whereas POI is improved by antibiotic treatment in the colon (Pohl et al., 2017). Besides, there is a big difference in the quantity and composition of the gut microbiota between the small and large intestines (Mowat and Agace, 2014; Martinez-Guryn et al., 2019), indicating the essential role of gut microbiota in the induction of POI. In the clinical setting, CRC patients with ileus have a lower α-diversity and a higher Firmicutes to Bacteroidetes ratio of gut microbiota compared with CRC patients without ileus (Jin et al., 2020). The relative abundance of Proteobacteria is high in CRC patients with ileus, whereas the relative abundances of Bacteroidetes, Firmicutes, and Fusobacterium are high in CRC patients without ileus. At the genus level, *Escherichia-Shigella*, *Ralstonia*, and *Veillonella* are significantly greater in the group with ileus than that without ileus. For POI, CRC patients with a low abundance of *Faecalibacterium* have a high risk of POI, and *Faecalibacterium* as a biomarker has an AUC value of 0.74 for the prediction of POI and an AUC value of 0.67 for the prediction of POI that occurs 6 months after discharge from hospital (Jin et al., 2020). Preoperative probiotic treatment improves bowel movement in the guinea pig with POI, probably by restoring the beneficial bacterial species *Bifidobacterium bifidum* and *Bifidobacterium longum* and increasing butyrate production (Shin et al., 2021). Moreover, a meta-analysis of 21 randomized controlled trials shows that prophylactic supplements of probiotics or synbiotics can effectively shorten the time of first flatus, first defecation, and first diet and reduce the incidence of POI in patients receiving GI cancer surgery (Tang et al., 2022). In contrast, preoperative stimulation of the efferent loop with probiotics does not affect the appearance of POI in patients undergoing loop ileostomy closure, which may be due to the previous existence of POI after colorectal cancer surgery (Rodriguez-Padilla et al., 2021). Nevertheless, perioperative gut microbiota modulation by supplementation of probiotics or synbiotics may be used to improve the recovery of GI motility after GI surgery (**Table 1**), but the underlying mechanisms and what changes in gut microbiota resulting from bowel preparation and surgery contribute to GI dysmotility remain to be elucidated.

### 5.4 Malabsorption

Complex anatomical GI reconstructions, such as gastrectomy, RYGB surgery, and pancreaticoduodenectomy, can lead to fat malabsorption, dumping syndrome, and vitamin deficiencies. Patients subjected to RYGB are at an increased risk of malabsorption resulting in trace element deficiency and osteopenia (Hammer, 2012; Billeter et al., 2015). Gut microbiota seems to be an important mediator in this process because FMT from RYGB-treated mice to germ-free mice without RYGB results in weight loss and decreased fat mass when compared with mice receiving FMT from sham surgery controls, potentially by regulating the production of SCFAs (Liou et al., 2013). The energy-reabsorbing potential of the gut microbiota, indicated by the Bacteroidetes to Firmicutes ratio, is decreased following laparoscopic sleeve gastrectomy (Damms-Machado et al., 2015), and gut microbiota can also play a significant role in the effects of RYGB on energy homeostasis (Chakravartty et al., 2015). The increased relative abundance of *Gammaproteobacteria* is closely associated with malabsorption after RYGB (Furet et al., 2010). Although malabsorption can be treated by vitamin supplementation and dietary modifications to adapt to the changed metabolism in many cases (Bland et al., 2016), the effect of the gut microbiota in the digestion and absorption after major surgery with GI reconstruction needs a further understanding, which will provide cues for development of therapies to accelerate postoperative recovery.

### 5.5 Cancer risk

The risk of colorectal cancer (CRC) may be increased after gastric bypass over time, whereas the risk of hormone-related cancers, including breast, endometrium, and prostate cancer, is associated with bariatric surgery (Derogar et al., 2013; Mackenzie et al., 2018). Besides, CRC risk is affected by sex and type of surgery. An increased risk of CRC is observed in males compared to females, especially 3 years or more after bariatric surgery, whereas CRC risk is decreased in females after RYGB but not sleeve gastrectomy (Hussan et al., 2022). Postoperative alteration of gut microbiota influences cancer risk and recurrence. Both decreases in absorptive GI mucosa and changes in the gut microbiota after bowel reconstruction contribute to disruption in the processing of bile acid (Flynn et al., 2015; Albaugh et al., 2019), which may expose colonocytes to more secondary bile acid, in particular deoxycholic acid (DCA), activating multiple signaling pathways including EGFR and Wnt in enterocytes that can lead to the development of CRC (Jia et al., 2018). Increased mucosal exposure to bile acid has been proposed to increase the risk of CRC after RYGB (Kant et al., 2014).

### 5.6 Cancer recurrence

**Figure 3:**
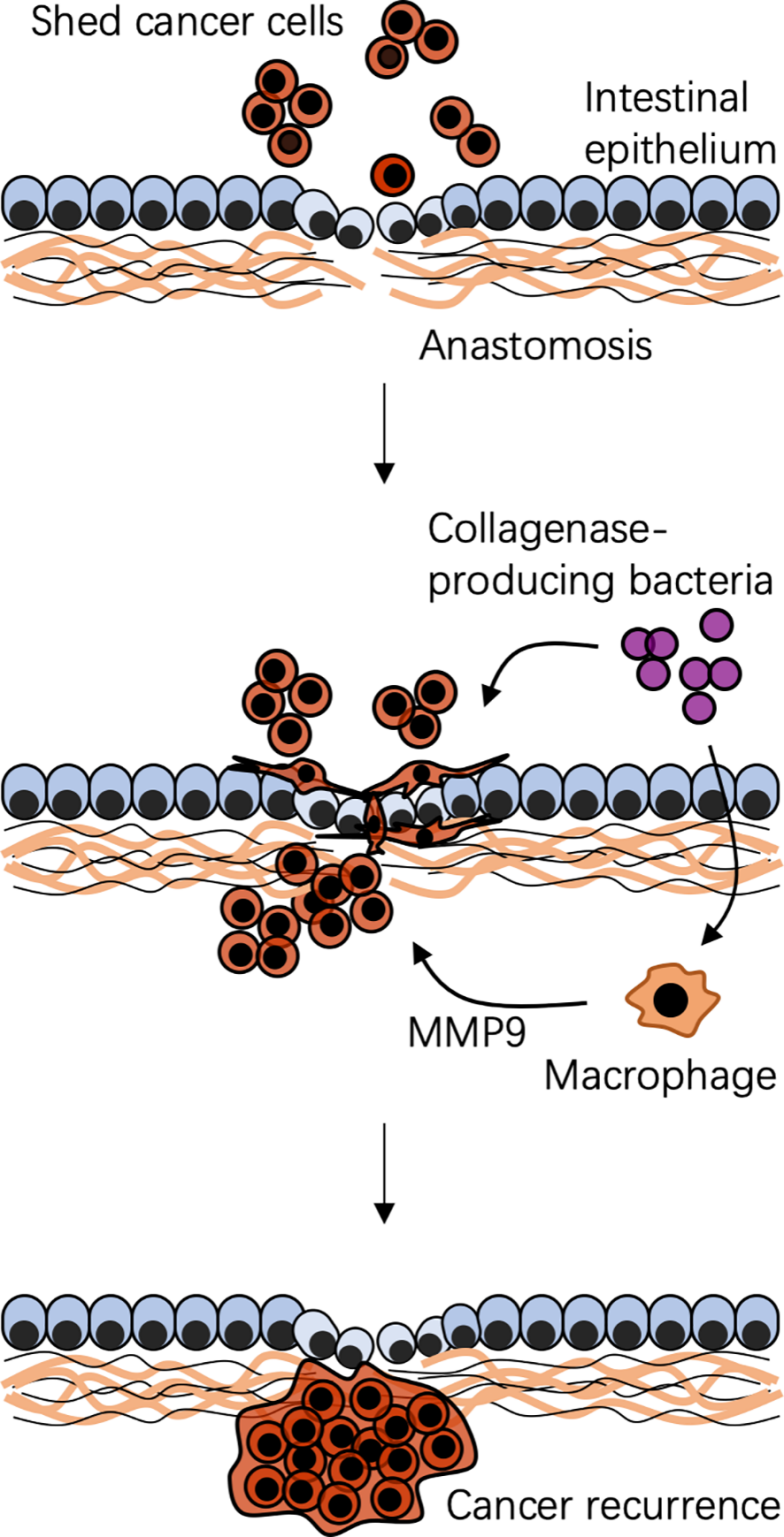
Possible mechanism of bacteria-associated colorectal cancer recurrence after surgical resection. A specific local context is created by surgical resection and reconstruction. Shed cancer cells occur during and after surgery. Context-dependent colonization of collagenase-producing bacteria, such as *Enterococcus faecalis*, cooperating with local macrophages, impairs anastomotic healing and promotes shed cancer cell proliferation, migration, and invasion, leading to cancer recurrence. MMP9, matrix metalloproteinase 9.

Gut microbiota also has been associated with CRC recurrence after surgery. Although patients undergoing surgery are often optimized with adjuvant or neoadjuvant chemoradiotherapy, up to one-third of them will get a postoperative CRC recurrence (Jeffery et al., 2007). In the traditional view, cancer recurrence is due to the postoperative blossom of shed tumor cells either at the surgical site or unknowingly present in the distal organs at the time of surgery. However, an opinion that the gut microbiota influences postoperative cancer recurrence is emerging (**Figure 3**).

Spatial-specific gut microbiota profiles link to CRC recurrence. The composition of gut microbiota at adjacent-tumor sites of patients with CRC recurrence is different from that in patients without CRC recurrence (Huo et al., 2022). At the phylum level, the relative abundance of Fusobacteria at adjacent tumor sites is much higher in patients with CRC recurrence than that in patients without CRC recurrence (Huo et al., 2022). Gut microbiota can serve as biomarkers to predict the risk of CRC recurrence. Besides, the postoperative gut microbiota can drive shed cancer cells to a migratory and aggressive phenotype, assisting them to escape immune surveillance to promote a postoperative recurrence. Through matrix metalloproteinases or the urokinase-plasminogen system, the collagenase-producing bacteria can bind to and degrade extracellular matrix, breakdown products of which are well known to promote proliferation, migration, and invasion of cancer cells (Shogan et al., 2015; He et al., 2016; Jacobson et al., 2020). *E. faecalis*, a high collagenase-producing strain, cooperating with macrophages reshapes colonic epithelial cells to a mesenchymal phenotype that is similar to the phenotype seen in mesenchymal transition, which is a fundamental biological process of tumor metastasis (Belogortseva et al., 2017). Indeed, colonization of collagenase-producing *E. faecalis* or *Proteus mirabilis* promotes shed cancer cells to form a tumor in mice on a high-fat diet undergoing a colon resection and anastomosis, which can be prevented by administration of a collagenase inhibitor (Gaines et al., 2020). Thus, gut microbiota-targeted strategies may be used to reduce the risk of CRC recurrence by eliminating these bacteria or inhibiting their collagenase activities.

## 6 Conclusion

Emerging evidence supports the role of gut microbiota in our understanding of postoperative complications. The causative role of gut microbiota in recovery from GI surgery needs to be further defined through in-depth research of the shift of bacterial phenotype, the interaction between gut microbiota and host, and their effects on the local microenvironment. Comprehensive knowledge of perioperative gut microbiota can provide a possibility for designing novel appropriate strategies for personalized bowel preparation. Fecal culture is limited to detail molecular information. Both before and after surgery, next-generation sequencing and phenotype analysis are necessary to serially track the composition and function of the bacterial community, which will advance the understanding of the gut microbiota and guide clinical care over the entire course of recovery. Resultant therapeutic innovations in the maintenance of gut microbiota and control of postoperative complications include methods to selectively kill pathogenic bacteria using narrow-spectrum antibiotics, antibody-labeled antibiotics, or engineered bacteriophages, and methods to restore gut microbiota using probiotics, prebiotics, bacterial ligands, or FMT. The molecular mechanistic understanding of the role of gut microbiota in postoperative complications will finally help to enhance recovery from GI surgery.

## Author contributions

ZZ managed the major literature research and wrote the first draft of the manuscript. YH, JT, WX, and WHZ managed literature research and reviewed the manuscript. WZ critically reviewed the manuscript and provided valuable discussions and criticisms. All authors contributed to the article and approved the submitted version.
